# Early supplemental feeding improves post-weaning growth restriction in lambs via the gastrointestinal-metabolic axis

**DOI:** 10.1128/aem.02421-25

**Published:** 2026-05-15

**Authors:** Lele Zhu, Mengmeng Wang, Xinyue Zhang, Tingting Dou, Heng Hua, Xinyue Tang, Xiaoyu Niu, Qifang Kou, Yulin Chen, Menghao Pan, Baohua Ma

**Affiliations:** 1 College of Veterinary Medicine, Northwest A&F University 12469, Yangling, China; 2 Wuzhong Hongsibao Tianyuan Fine-Breed Sheep Farming & Propagation Co., Ltd, Wuzhong, China; 3 College of Animal Science and Technology, Northwest A&F University 12469, Yangling, China; Universita degli Studi di Napoli Federico II, Portici, Italy

**Keywords:** early supplemental feeding, rumen microbiota, intestine, metabolism, weaning-induced growth restriction, lambs

## Abstract

The weaning period often induces growth restriction in lambs due to gastrointestinal immaturity and dietary transition. Although early supplemental feeding (ESF) shows promise, its systemic mechanisms via the rumen microbiota-gut-metabolism axis remain incompletely understood. Sixty Tan lambs were assigned to a control group (suckling only), Trial Group I (free-access ESF from 10 days), and Trial Group II (free-access + forced isolation from 15 days). Growth performance, rumen fermentation, intestinal morphology, serum metabolites, and rumen microbiota were comprehensively analyzed. The supplemental feeding regimen in Trial Group I significantly improved body weight, average daily gain, and body size before and after weaning. Rumen butyrate and acetate concentrations increased markedly, with enrichment of beneficial microbiota (*Bifidobacteriaceae* and *Sharpea*). Intestinal villus height and villus-to-crypt ratio were enhanced in the jejunum and ileum. Regarding serum metabolism, lambs in both supplemental feeding groups showed significantly increased levels of albumin and urea and decreased total cholesterol. The activities of antioxidant enzymes (catalase, superoxide dismutase, and glutathione peroxidase) and the levels of oxidative products (malondialdehyde and hydrogen peroxide) in the serum of lambs from both supplemental feeding groups were significantly higher than those in the control group. Correlation analysis revealed positive associations between beneficial microbiota, fermentation parameters, intestinal development, and growth performance. ESF alleviates post-weaning growth restriction by remodeling the rumen microbiota, enhancing volatile fatty acid production, improving intestinal development, and optimizing host nutrient utilization. The free-access ESF regimen is recommended for efficient lamb production.

**IMPORTANCE:**

This study reveals that different regimens of early supplemental feeding differentially alleviate post-weaning growth restriction in lambs by distinctly remodeling the host’s “rumen microbiota-gut-metabolism axis.” The optimized regimen enriches beneficial rumen microbiota (e.g., *Bifidobacteriaceae* and *Sharpea*), increases key metabolite production (e.g., butyrate), and synergistically improves intestinal development and host protein/lipid metabolism, thereby elucidating the mechanism of growth promotion via microbe-host interactions. These findings advance the understanding of “nutrition-microbe-host” crosstalk in young ruminants and provide a theoretical framework for precision nutrition through microbiome modulation. The identified key microbes and metabolic functions offer potential targets for developing eco-friendly feed additives, aiding in reducing antibiotic reliance and promoting sustainable lamb production.

## INTRODUCTION

In intensive farming systems, the weaning period in lambs (typically 3–8 weeks of age) is often characterized by severe growth retardation, increased diarrhea incidence, and elevated mortality rates, significantly constraining production efficiency (1). Early growth impairment not only affects immediate weight gain but also exerts persistent negative effects on lifetime production performance (2, 3). The underlying physiological mechanism primarily involves nutritional stress and physiological adaptation imbalances during the transition from liquid milk to solid feed (4). Research indicates that the first 2 months postpartum represent a critical window for rumen development in lambs, during which nutritional management directly influences papillary growth, microbial community establishment, and metabolic function maturation (5, 6). Traditional weaning practices typically occur at 60–90 days of age, but recent studies suggest that scientifically managed early weaning combined with precise nutritional interventions can alleviate weaning stress and promote early rumen functional maturation, establishing a foundation for lifelong growth (7, 8).

Early supplemental feeding (ESF), as a targeted nutritional intervention, refers to the management technique of proactively providing highly digestible, nutrient-dense starter feed during the suckling period (usually starting at 10–15 days of age) to guide lambs in consuming solid feed, thereby facilitating a smooth transition to complete weaning (9). This technique leverages the physiological development characteristics of lambs, capitalizing on the critical period around 10 days of age when feeding behavior begins to emerge, using exogenous nutritional intervention to promote digestive system development, particularly of the rumen (10, 11). ESF can drive the establishment of ruminal microbes and fermentation functions through increased solid feed intake, yielding persistent benefits on post-weaning growth performance (12, 13). ESF not only affects short-term growth performance but also exerts persistent effects on subsequent development (14). During the early fattening period (66–120 days), lambs exhibited significant compensatory growth effects, with average daily gain significantly higher than traditionally reared groups (15). This compensatory growth phenomenon confirms the “programming effect” of early nutritional intervention on lamb metabolic pathways, meaning that appropriate nutritional stimulation during early life can optimize metabolic pathways and produce lasting physiological effects (10, 16).

Rumen functional maturation is a core aspect and physiological foundation of lamb growth and development (17, 18). The neonatal lamb rumen is relatively primitive and underdeveloped, with incomplete structure and function (5). However, the transition from the suckling period to post-weaning solid feed consumption represents a “critical window period” for the rumen to undergo rapid morphological development and functional maturation (19). The success of this process directly determines whether lambs can effectively utilize solid feed after weaning and avoid growth stagnation or weight loss (20). The establishment and succession of the rumen microbial community and rumen development are mutually reinforcing, together constituting the two cornerstones of lamb digestive capacity (21). Microbial groups, including bacteria, archaea, protozoa, and fungi, form complex interaction networks within the rumen, responsible for key physiological functions such as cellulose and hemicellulose degradation, starch and protein fermentation, and vitamin synthesis (22). The volatile fatty acids (VFAs) they metabolically produce are not only the host’s primary energy source but also key signaling molecules stimulating rumen epithelial development (23). The small intestine, as the primary site for nutrient digestion and absorption and a crucial barrier, directly determines the final utilization efficiency of nutrients by lambs and is another decisive factor affecting lamb growth performance (24). Unlike the rumen, which develops later, the lamb small intestine is relatively mature at birth and capable of efficient digestion and absorption of liquid milk (25, 26). Fats, lactose, and immunoglobulins in milk are primarily decomposed and absorbed here, providing energy and passive immune protection for newborn lambs. However, this physiological state adapted to liquid diets must undergo functional transformation toward adapting to complex solid diets with weaning and the introduction of solid feed (27). This transformation process involves profound remodeling of small intestinal morphological structure and digestive function (28). Ideal lamb growth depends on the coordinated development and efficient integration of rumen fermentation function and small intestinal absorption function.

Although ESF has demonstrated significant effectiveness in practice, its intrinsic biological mechanisms—particularly how it coordinately promotes lamb growth by regulating rumen microbial community succession, gastrointestinal morphological development, and host systemic metabolism through the “microbiota-gut-metabolism axis” have not been systematically elucidated. Existing research mostly focuses on growth phenotypes and basic fermentation parameters, lacking in-depth analysis of interaction networks between multiple systems. Therefore, this study used Tan lambs as a model to systematically evaluate the effects of different supplemental feeding regimens on lamb growth performance, rumen fermentation, intestinal tissue morphology, blood metabolic indicators, and oxidative stress status, combined with rumen microbiota analysis. The aim was to reveal the mechanism by which ESF improves post-weaning growth restriction in lambs through the “rumen microbiota-gut development-host metabolism axis,” providing a theoretical basis and practical guidance for optimizing lamb rearing strategies.

## RESULTS

### Early supplemental feeding effectively improves lamb growth performance

**Fig 1:**
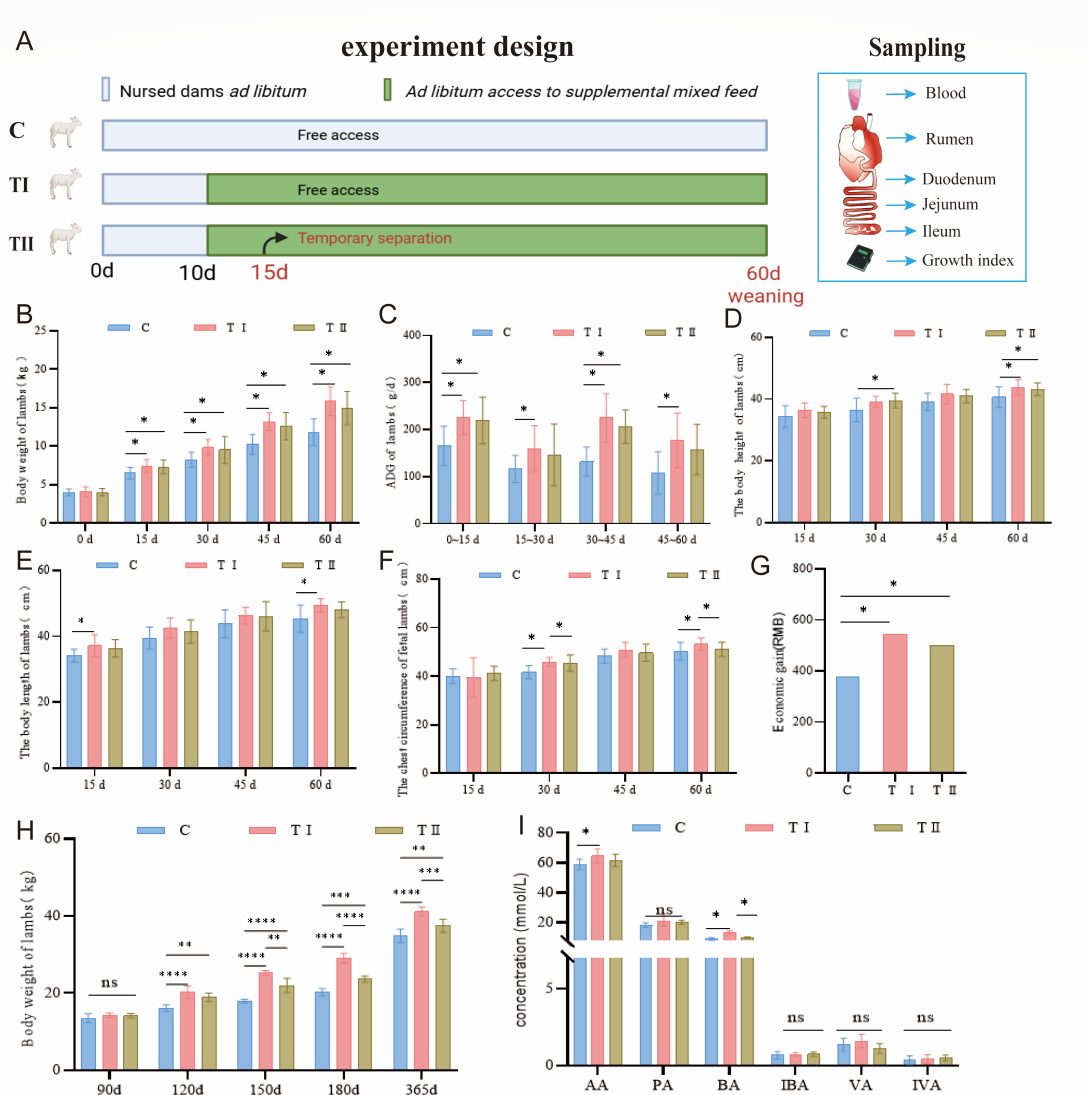
Effects of different early supplemental feeding regimens on lamb growth performance and rumen fermentation parameters at 60 days of age. (**A**) Schematic diagram of the experimental design. Lambs in the C group suckled freely from their mothers. Lambs in the TI group suckled freely from their mothers after birth and began to have free access to a supplemental mixed feed from 10 days of age, using a creep feeding system, where lambs could move freely, but ewes were excluded. Lambs in group TII were reared with *ad libitum* suckling from their dams after birth, began *ad libitum* access to a supplemental mixed feed from 10 days of age, and were forcibly separated from their dams in the creep feeding area with *ad libitum* access to the supplemental mixed feed daily from 9:00 a.m. to 5:00 p.m. starting at 15 days of age. Blue: nursed dams *ad libitum*. Green: *ad libitum* access to supplemental mixed feed. (**B**) Body weight of lambs under different regimens. (**C**) Average daily gain (ADG) of lambs under different regimens. (**D**) Body height of lambs. (**E**) Body length of lambs. (**F**) Chest circumference of lambs. (**G**) Economic benefits of lamb rearing, expressed in RMB. (**H**) Monitoring the body weight of lambs post-weaning under different regimens. (**I**) Relative proportions of volatile fatty acids (acetate, propionate, isobutyrate, butyrate, isovalerate, and valerate). Statistical comparisons were conducted using Student’s *t*-test. Data are presented as mean ± SEM. Significance levels: n.s., *P* > 0.05; *P < 0.05; **P < 0.01; ***P < 0.001; and ****P < 0.0001.

To systematically evaluate the promoting effect of early supplemental feeding on lamb growth and development, we first analyzed growth performance. During the lamb rearing period, initial body weight did not differ significantly among groups, and body weight showed an increasing trend with age. By 60 days of age, body weight of trial group lambs was significantly higher than that of the control group (*P* < 0.05) (Fig. 1B). During the 0–15 and 30–45 day periods, the daily gain of trial group lambs was significantly higher than that of the control group (*P* < 0.05), reaching over 200 g/day at maximum. Additionally, the daily gain of Trial Group I (TI group) during the 15–30 and 45–60 day periods was also significantly higher than that of the control group (*P* < 0.05) (Fig. 1C). Body measurement results showed that body height of TI and TII (Trial Group II) lambs at 30 and 60 days of age was significantly higher than that of the control group (*P* < 0.05) (Fig. 1D). At 15 days of age, body length of both TI and TII groups was significantly higher than that of the control group (*P* < 0.05); by 30 and 60 days of age, body length of the TI group remained significantly higher than that of the control group (*P* < 0.05) (Fig. 1E). At 30 and 45 days of age, chest width of trial group lambs was significantly higher than that of the control group (*P* < 0.05); at 60 days of age, chest width, chest depth, and chest circumference of the TI group remained significantly higher than those of the control group (*P* < 0.05), while no significant differences were observed in the TII group (Fig. 1F; Fig. S1E and F). Post-weaning trial group body weight at 90 days was significantly higher than that of the control group lambs (*P* < 0.05); at 120, 150, and 180 days, body weight of Trial Group I lambs remained significantly higher than that of the control group (*P* < 0.05) (Fig. 1H). Economic benefit analysis showed that the economic return per lamb in the supplemental feeding groups increased by over 400 yuan compared to the control group, with the TI group supplemental feeding regimen showing the best economic benefit (Fig. 1G). The results indicate that early supplemental feeding improves pre-weaning lamb growth performance while alleviating post-weaning growth restriction.

### Early supplemental feeding optimizes lamb rumen fermentation

Given that rumen fermentation is a core aspect of nutrient utilization in ruminants, we further investigated the effects of early supplemental feeding on rumen fermentation parameters in 60-day-old lambs. Results showed that rumen pH and ammonia nitrogen (NH_3_-N) concentration were slightly higher in the control group than in the trial groups, but the differences were not significant (*P* > 0.05) (Fig. S1A and B). Butyrate concentration in the TI group was significantly higher than in the control and TII groups (*P* < 0.05), and acetate concentration was also significantly higher than in the control group (*P* < 0.05) (Fig. 1I). Total volatile fatty acid (TVFA) concentration and the acetate-to-propionate ratio did not differ significantly among groups (*P* > 0.05) (Fig. S1C and D). These results suggest that the TI group’s supplemental feeding regimen effectively optimizes the rumen fermentation process and improves energy substance production efficiency.

### Early supplemental feeding improves lamb intestinal morphology

**Fig 2:**
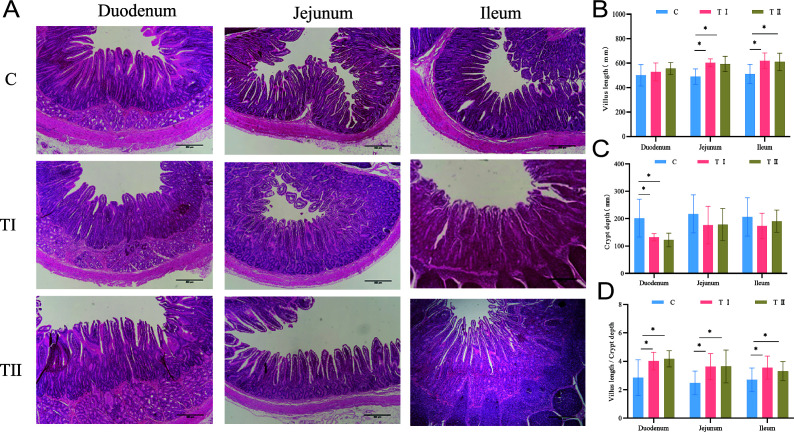
Effects of different early feeding regimens on the intestinal development of lambs. (**A**) Schematic of intestinal tissue sections from lambs at 60 days under different regimens (40× magnification). (**B**) Villus height in the duodenum, jejunum, and ileum. (**C**) Crypt depth in the duodenum, jejunum, and ileum. (**D**) Villus height to crypt depth ratio (V/C ratio). Significance levels: n.s., *P* > 0.05; *P < 0.05.

The intestine is the primary site for nutrient absorption, and its morphological structure directly determines absorption efficiency. Observation of small intestinal tissue sections from 60-day-old lambs showed that villus height was significantly greater in TI and TII groups than in the control group, with a tighter and more orderly arrangement. Villus height in the jejunum and ileum was significantly higher in the trial groups than in the control group (*P* < 0.05), while no significant difference in duodenal villus height was observed among groups (Fig. 2A and B). Duodenal crypt depth in the control group was significantly higher than in the trial groups (*P* < 0.05) (Fig. 2A and C), suggesting lower intestinal maturity or mild stress. The villus height to crypt depth ratio in the duodenum, jejunum, and ileum was significantly higher in the trial groups than in the control group (*P* < 0.05) (Fig. 2D). In summary, the TI group supplemental feeding regimen significantly promoted small intestinal villus development, optimized intestinal structure, and enhanced nutrient absorption capacity.

### Early supplemental feeding alters lamb material metabolism

**Fig 3:**
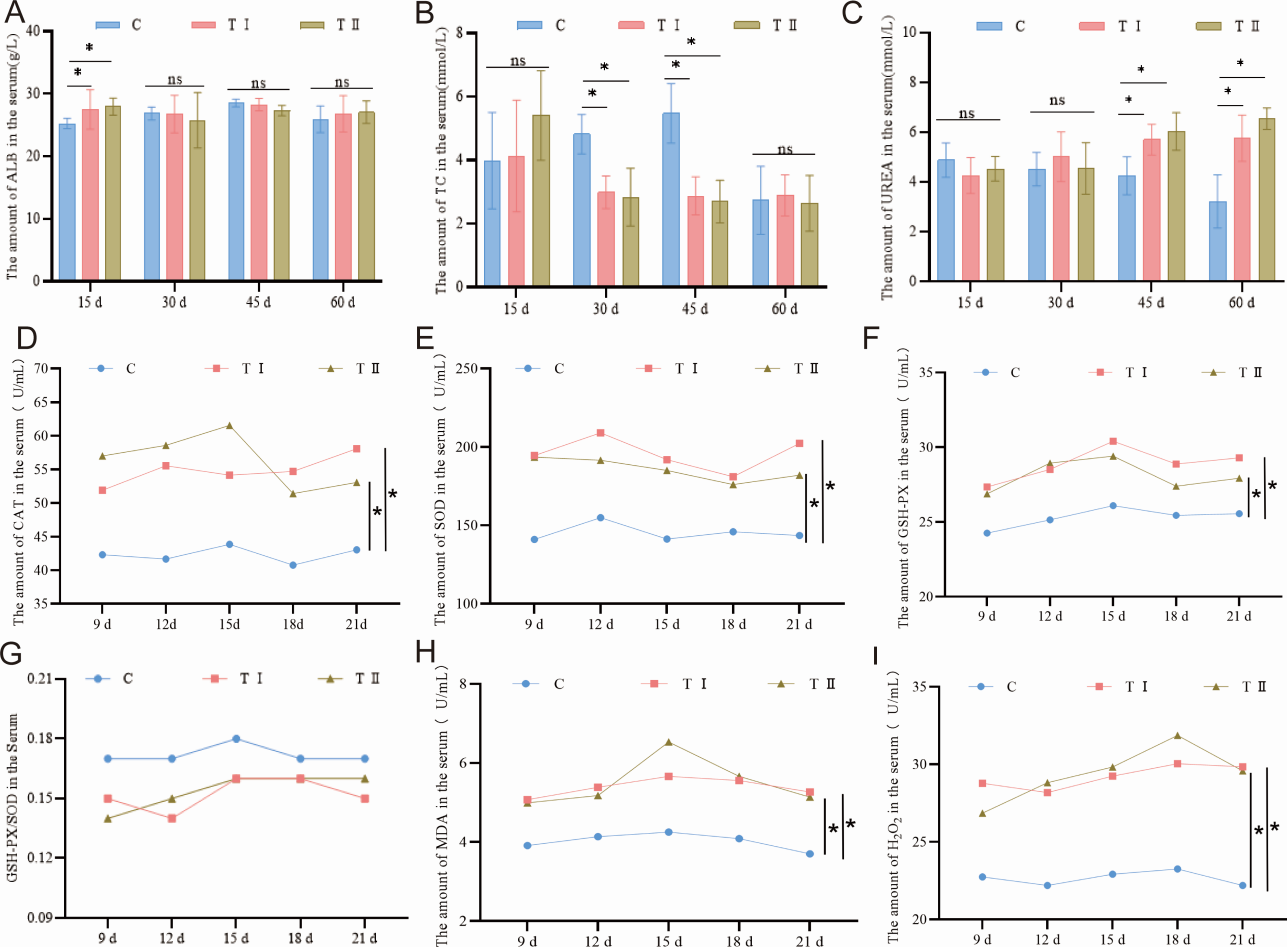
Effects of different early feeding regimens on serum biochemistry, oxidative stress, and antioxidant capacity in lambs. (**A**) Serum ALB concentration. (**B**) Serum TC concentration. (**C**) Serum UREA concentration. (**D**) Serum catalase (CAT) activity. (**E**) Serum superoxide dismutase (SOD) activity. (**F**) Serum glutathione peroxidase (GSH-PX) activity. (**G**) GSH-PX/SOD ratio. (**H**) Serum malondialdehyde (MDA) concentration. (**I**) Serum hydrogen peroxide (H_2_O_2_) concentration. Significance levels: n.s., *P* > 0.05; *P < 0.05.

To reveal the intrinsic metabolic mechanism by which early supplemental feeding promotes growth, we determined serum biochemical indicators. No significant differences were found in growth hormone (GH) and insulin-like growth factor I (IGF-I) levels among groups (*P* > 0.05), indicating that the growth-promoting effect of early supplemental feeding is not mediated through the GH-IGF-I axis (Fig. S2A and B). Throughout the trial period, serum total protein (TP), triglyceride (TG), and glucose levels showed no significant differences among groups (*P* > 0.05) (Fig. S2C through E). At 15 days of age, serum albumin (ALB) content in TI and TII groups was significantly higher than in the control group (*P* < 0.05), reflecting more active protein anabolic metabolism (Fig. 3A). At 30 and 45 days of age, serum total cholesterol (TC) level in the control group was significantly higher than in the trial groups (*P* < 0.05), suggesting higher lipid utilization efficiency in the supplemental feeding groups (Fig. 3B). At 45 and 60 days of age, serum urea (UREA) content in the trial groups was significantly higher than in the control group (*P* < 0.05), indicating more vigorous protein metabolic turnover (Fig. 3C). Early supplemental feeding affects growth performance by optimizing the nutrient metabolism of the lamb body rather than altering the GH-IGF-I endocrine axis.

### Early supplemental feeding affects lamb oxidative stress and antioxidant capacity

Animal growth performance is closely related to the body’s oxidative stress status; therefore, we evaluated the antioxidant capacity of lambs. Analysis of serum antioxidant indicators at 9, 12, 15, 18, and 21 days of age showed that catalase (CAT), SOD, and GSH-PX in the trial groups were significantly higher than those in the control group (*P* < 0.05) (Fig. 3D through F). The GSH-PX/SOD value in group C tended to be higher compared to the other two groups (*P* > 0.05) (Fig. 3G). Malondialdehyde (MDA) and hydrogen peroxide (H_2_O_2_) contents in the trial groups were slightly higher than those in the control group (*P* < 0.05) (Fig. 3H and I), while the CAT/SOD value showed no significant difference among groups (*P* > 0.05) (Fig. S2F). Early supplementary feeding enhanced the synergistic function of the body’s antioxidant enzyme system.

### Early supplemental feeding alters lamb rumen microbiota

**Fig 4:**
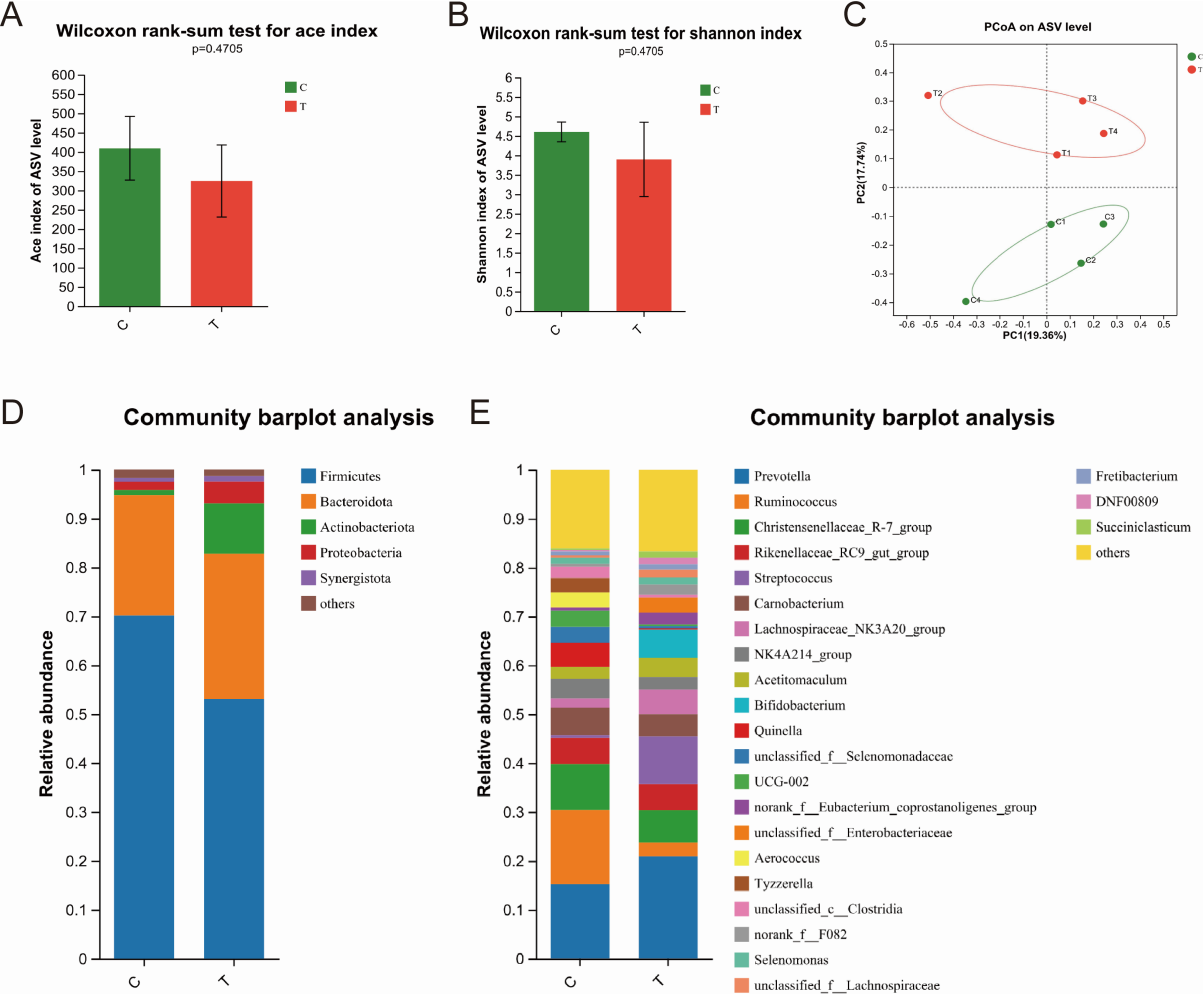
Effects of different early feeding regimens on ruminal microbial changes in 60-day-old lambs. (**A**) ACE index at the amplicon sequence variant (ASV) level. (**B**) Shannon index at the ASV level. (**C**) PCoA at the ASV level. (**D**) Composition of rumen bacteria at the phylum level. (**E**) Composition of rumen bacteria at the genus level.

**Fig 5:**
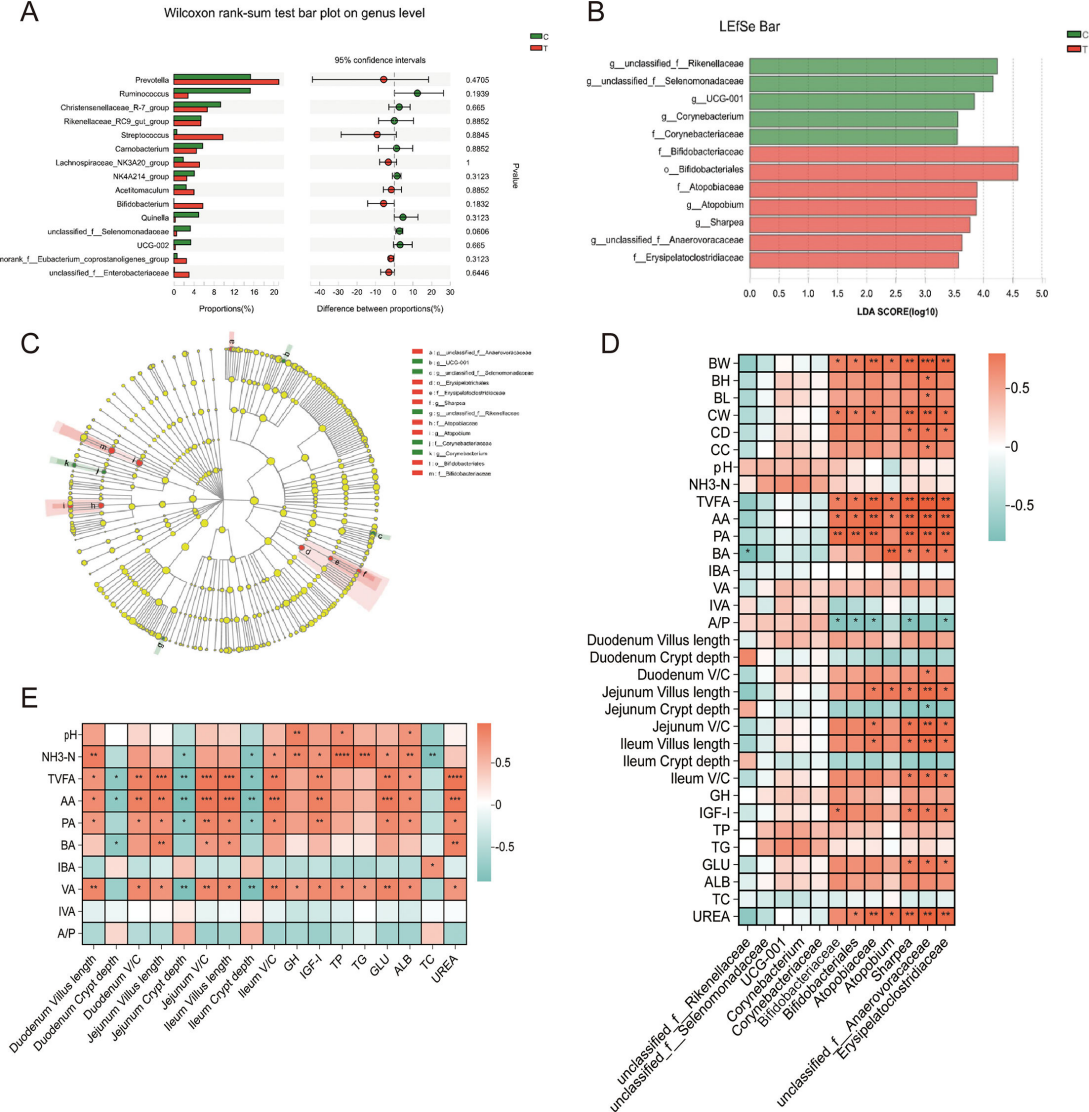
Differential ruminal microbiota and correlation analysis in lambs under different early feeding regimens. (**A**) Top 15 differentially abundant microbiota at the genus level between the C and T groups. (**B**) LEfSe identifying significantly enriched bacterial taxa between the C and T groups using an all-against-all multiclass comparison strategy (LDA score > 3). (**C**) Cladogram between the C and T groups. (**D**) Spearman correlation analysis between differentially abundant ruminal microbiota at the species level and growth indices, fermentation parameters, intestinal development indices, and serum metabolites in the C vs T groups. (**E**) Spearman correlation analysis between fermentation parameters and intestinal development indices and serum metabolites in the C vs T groups. The color scale represents Spearman’s correlation coefficients (ρ): red indicates a positive correlation (variables increase or decrease together), whereas blue indicates a negative correlation (variables change in opposite directions). Correlations reflect associations between variables rather than direct correlations with experimental groups. Significance levels: n.s., *P* > 0.05; *P < 0.05; **P < 0.01; ***P < 0.001; and ****P < 0.0001.

Rumen microbes play a key role in the decomposition and transformation of nutrients, and changes in their community structure may be the underlying reason for the effects of supplemental feeding. Analysis of 16S rRNA gene sequences from the rumen of 60-day-old lambs showed that there were no significant differences in microbial richness (abundance-based coverage estimator [ACE] index) or diversity (Shannon index) between the control (C) and TI groups (*P* > 0.05) (Fig. 4A and B). Principal coordinates analysis (PCoA) plot showed clear separation of rumen microbiota between the two groups, indicating significant differences in microbial structure between C and T groups (*P* < 0.05) (Fig. 4C). At the phylum level, rumen microbes in both groups were dominated by Firmicutes and Bacteroidota, followed by Actinobacteria, Proteobacteria, and Synergistota (Fig. 4D). At the genus level, *Prevotella* was the dominant genus (Fig. 4E). Further analysis showed no significant differences in the relative abundance of the top 15 bacterial groups at the genus level (*P* > 0.05) (Fig. 5A). Cladogram and linear discriminant analysis (LDA) effect size (LEfSe) analysis (LDA > 3.5) showed that the dominant microbiota in the C group were *unclassified_f__Rikenellaceae*, *unclassified_f__Selenomonadaceae*, *UCG-001, Corynebacterium*, and *Corynebacteriaceae*; the dominant microbiota in TI were *Bifidobacteriaceae*, *Bifidobacteriales*, *Atopobiaceae*, *Atopobium*, *Sharpea*, *unclassified_f__Anaerovoracaceae*, and *Erysipelatoclostridiaceae* (Fig. 5B and C).

### Correlations among rumen differential bacteria, fermentation parameters, intestinal development, serum metabolites, and growth performance

To integrate the above findings and establish causal links from microbes to host phenotypes, we finally performed correlation analysis of multi-omics indicators. The correlation analysis was performed across all individual samples, and the color scale represents Spearman’s correlation coefficients between variables rather than direct associations with treatment groups. Color scale represents Spearman’s correlation coefficients (ρ): red indicates a positive correlation (variables increase or decrease together), whereas blue indicates a negative correlation (variables change in opposite directions). Although the TI group did not significantly alter the overall diversity of rumen microbiota, it induced significant restructuring of the microbial community and enriched beneficial genera such as *Bifidobacteriaceae* and *Atopobium*. Correlation analysis showed that the enriched microbiota in the TI group was significantly positively correlated with total VFA, acetate, propionate, butyrate, insulin-like growth factor-I, and urea (*P* < 0.05). Among them, *Bifidobacteriaceae* and *Sharpea*, as efficient carbohydrate-fermenting bacteria, were significantly positively correlated with rumen butyrate and acetate concentrations (*P* < 0.05). Furthermore, the relative abundance of these beneficial bacterial groups was also significantly positively correlated with lamb body weight, chest circumference, jejunal villus length, and ileal villus length (*P* < 0.05) (Fig. 5D). Rumen fermentation parameters were significantly positively correlated with intestinal villus height and significantly negatively correlated with crypt depth (*P* < 0.05). Meanwhile, total VFA, acetate, propionate, butyrate, etc., were also significantly positively correlated with serum IGF-I, glucose, albumin, and urea levels (*P* < 0.05) (Fig. 5E). These results indicate that early supplemental feeding, by regulating rumen microbial composition, optimizes the fermentation process, promotes intestinal development and nutrient metabolism, and ultimately enhances lamb growth performance and body size development, while alleviating post-weaning growth restriction.

## DISCUSSION

This study found that early supplementation (especially in Group TI) significantly increased the ruminal concentrations of butyrate and acetate (*P* < 0.05) and enriched beneficial microorganisms, including *Bifidobacteriaceae* and *Sharpea. Bifidobacteriaceae* are not only important carbohydrate-fermenting bacteria, but recent research has increasingly highlighted their potential as targets for prebiotic/probiotic interventions to improve ruminant health (29). Previous studies have indicated that directly fed microbes, such as bifidobacteria, can stabilize the gastrointestinal tract microbiota in early-weaned ruminants by competitively excluding pathogens and modulating local immunity (30). Other research has reported that core functional taxa like *Sharpea* play a critical role in lactate utilization and butyrate production pathways, and their abundance is crucial for predicting ruminal metabolic homeostasis (31). These microorganisms are known as efficient carbohydrate fermenters, capable of degrading plant polysaccharides to produce short-chain fatty acids (SCFAs), particularly butyrate and acetate. Butyrate serves not only as a vital energy source but also as a key signaling molecule regulating the proliferation and differentiation of rumen epithelial cells (32). An increase in its content is closely associated with the development of ruminal papillae (33). This finding aligns with previous research indicating that solid feed intake drives the enrichment of fiber-degrading and butyrate-producing bacteria, thereby establishing the foundation for ruminal functional maturation (34, 35). Our correlation analysis further confirmed that the relative abundances of *Bifidobacteriaceae* and *Sharpea* were significantly positively correlated with ruminal butyrate and acetate concentrations, as well as with lamb body weight and body size indices (*P* < 0.05). Consistently, prior studies have also demonstrated that early supplementation can optimize ruminal fermentation function by altering solid feed intake to promote the enrichment of fiber-degrading and butyrate-producing bacteria (36). Furthermore, the significant difference in rumen microbial structure (PCoA analysis) but no significant change in diversity in the supplemental feeding groups in this study suggests that the role of early supplemental feeding is mainly reflected in regulating the abundance of specific functional bacterial groups rather than increasing overall microbial diversity. The early gastrointestinal microbial colonization in lambs has a clear “window period,” and the intervention of solid feed can guide the microbial community structure toward a direction more conducive to fiber digestion and SCFA production (37).

This study revealed a crucial finding: the benefits of early supplementation transcend ruminal development, significantly enhancing the morphology of the distal small intestine. Specifically, early supplementation led to significant increases in villus height and the villus length-to-crypt depth ratio (V/C ratio) in the jejunum and ileum (*P* < 0.05), alongside a reduction in duodenal crypt depth. Since villus architecture directly dictates the surface area available for nutrient absorption, and the V/C ratio serves as a key metric for intestinal functional maturity and overall health, these morphological improvements underscore the systemic impact of the nutritional intervention (38). More importantly, our correlation analysis revealed the existence of a “rumen-gut axis”: the concentrations of total volatile fatty acids, acetate, and butyrate in the rumen showed significant positive correlations with villus height in the jejunum and ileum (*P* < 0.05). This indicates that early supplementation promotes the proliferation of beneficial microorganisms and the generation of their metabolites (such as SCFAs), thereby providing both essential energy substrates and signaling stimuli for intestinal development. Our finding of a close association between butyrate and intestinal villus development is particularly noteworthy. Previous discussions on the benefits of postbiotics (e.g., bacterial metabolites) for gut health have highlighted that butyrate serves as the preferred energy source for colonocytes and exerts anti-inflammatory effects while promoting epithelial barrier repair through the inhibition of histone deacetylase (39). This suggests that the phenomenon observed in lambs may be underpinned by an evolutionarily conserved, microbial metabolite-mediated regulatory mechanism for intestinal epithelium that exists across mammalian species. Supporting this notion, previous studies have shown that dietary interventions capable of altering rumen fermentation patterns can modify ruminal microbial communities and metabolic outputs, which in turn influence host physiological responses and potentially affect microbial ecosystems beyond the rumen (40–42). These findings collectively suggest that modulation of rumen fermentation status may exert systemic effects extending to distal regions of the gastrointestinal tract. Furthermore, the rapid development of the rumen post-weaning appears interconnected with morphological evolution in the intestines, suggesting that ruminal fermentation products induced by solid feed may indirectly influence the development of distal intestinal segments via circulatory or neuroendocrine pathways (43, 44). Thus, the improvement in intestinal morphology observed in our study can be attributed to the coordinated interactions along the “rumen-gut axis.”

At the host metabolic level, while early supplementation did not significantly alter the concentrations of GH or IGF-I, it induced a distinct metabolic shift characterized by significantly elevated serum ALB and UREA (*P* < 0.05), coupled with reduced TC. This pattern aligns with the concept, observed in calf studies, that early-life gastrointestinal health is a critical determinant of nutrient partitioning and the “immune-metabolic” trade-off (45, 46). Collectively, these metabolic alterations point to an underlying growth-promoting mechanism: the rise in ALB and UREA indicates enhanced whole-body protein anabolism. Specifically, the elevated UREA, being a terminal product of protein catabolism, likely signifies greater dietary protein intake and degradation, as well as accelerated amino acid turnover. This is consistent with the increased consumption of starter feed by supplemented lambs and the consequent augmentation of ruminal microbial protein synthesis (47, 48). The decrease in TC suggests higher lipid utilization efficiency in supplemental feeding group lambs, possibly directing more energy toward tissue growth rather than fat storage (49). These results collectively indicate that the growth-promoting effect of early supplemental feeding is not achieved through the classic endocrine growth axis (GH-IGF-I) but by optimizing the body’s nutrient allocation and utilization efficiency, that is, driving the metabolic state from mainly “maintenance” to mainly “growth.” It is noteworthy that the activities of antioxidant enzymes (CAT, SOD, and GSH-PX) in the lambs of the supplemented groups were significantly higher than those in the control group (*P* < 0.05), indicating that their antioxidant defense system was activated earlier. Although the serum levels of H_₂_O_₂_ and MDA were also slightly elevated, suggesting an increased oxidative burden due to enhanced metabolism, the supplemented lambs effectively maintained their redox homeostasis through the synergistic action of the enhanced enzyme system. This adaptive change indicates that early supplementary feeding not only supports more vigorous material metabolism but also enhances the physiological resilience of lambs to environmental stressors such as weaning. This finding is in line with the study by Natalello et al. (50), which suggests that rational nutritional intervention helps maintain the dynamic balance between the oxidative and antioxidant systems in lambs during periods of rapid growth, thereby providing a stable internal environment for efficient growth.

Our analysis of economic benefits demonstrated that the Group TI (free creep feeding) regimen enhanced profit per lamb by more than 400 RMB, which is largely attributable to its superior cost-effectiveness. This strategy effectively circumvented the potential for added managerial expenses and stress inherent in the forced feeding approach of Group TII, while simultaneously maximizing growth performance via continuous access to a high-quality supplemental diet. The findings of this study are primarily based on 16S rRNA gene sequencing and correlation analyses, providing strong correlative evidence that early supplementary feeding exerts its effects through the “microbiota-gut-metabolism axis.” However, to directly validate the causal roles of specific microorganisms (such as *Bifidobacteriaceae* and *Sharpea*) and their metabolites (e.g., butyrate) in this process, future research should integrate metagenomics to elucidate the functional gene repertoire of the microbial community, combined with metabolomics (particularly targeted short-chain fatty acid metabolomics) to quantitatively track the dynamic changes of both host-derived and microbial-derived metabolites.

**Fig 6:**
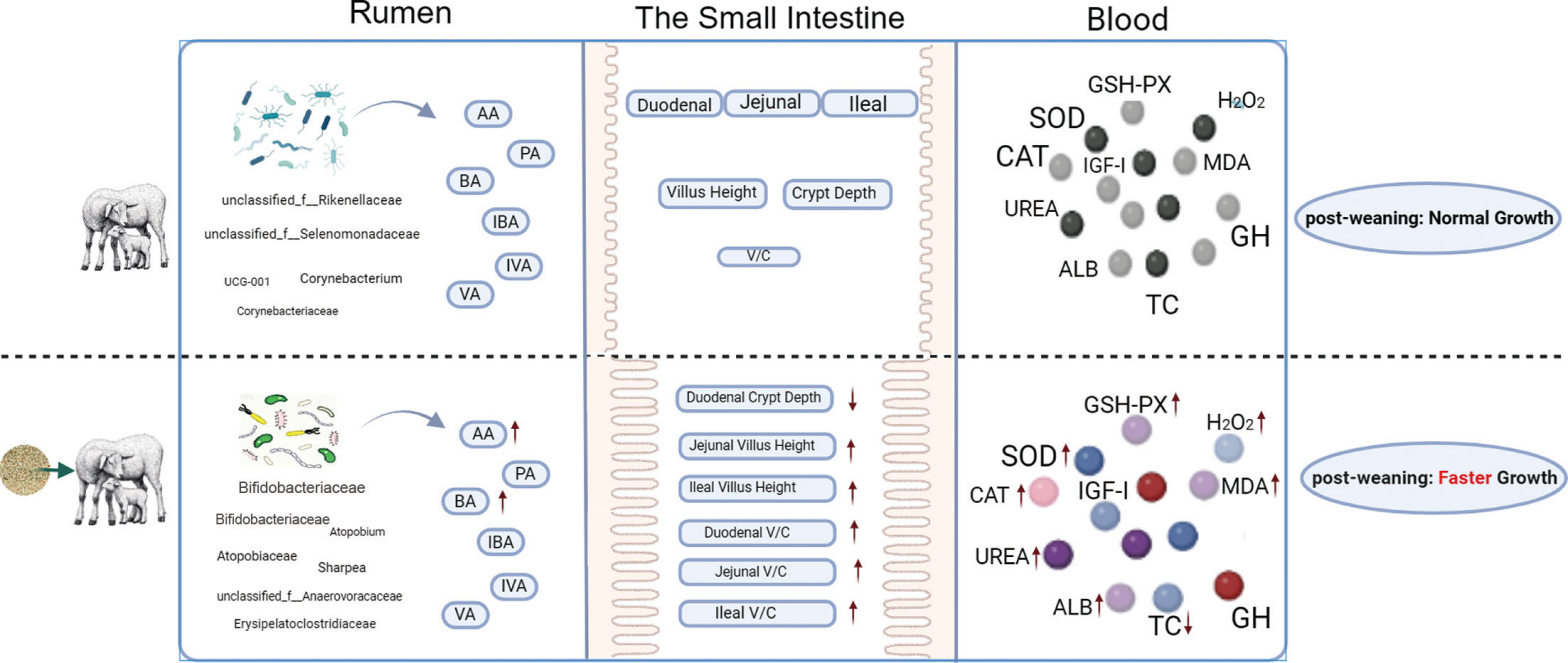
Proposed mechanism underlying the effects of early feeding (T group regimen). Early supplementary feeding (the T group regimen) enriched beneficial microorganisms such as *Bifidobacteriaceae* and *Sharpea*, thereby optimizing the ruminal microbial structure. These beneficial microbiota directly promoted the production of acetate and butyrate, providing a more abundant energy source for the lambs. The optimized ruminal fermentation environment and its associated signals synergistically promoted small intestinal villus development and intestinal structural maturation (evidenced by increased villus height and V/C ratio), consequently enhancing nutrient absorption capacity. This was manifested as more efficient protein synthesis (increased serum ALB and UREA levels) and more effective lipid utilization (decreased serum TC levels). Concurrently, the body effectively maintained redox homeostasis through an enhanced antioxidant defense system (characterized by increased activities of antioxidant enzymes CAT, SOD, and GSH-PX, along with slightly elevated levels of oxidative products MDA and H_2_O_2_), providing a robust physiological internal environment for efficient growth. Ultimately, this cascade of effects directed ingested nutrients more efficiently toward growth deposition, comprehensively enhancing the growth performance and body size development of the lambs and successfully alleviating post-weaning growth impairment.

Early supplemental feeding, especially the TI group regimen, can significantly optimize the physiological metabolism and development status of lambs by reshaping the rumen microbial community structure. This regimen effectively enhances the production of acetate and butyrate in the rumen by enriching beneficial microorganisms such as *Bifidobacteriaceae* and *Sharpea*, providing a more abundant energy source for the host. This optimized rumen fermentation environment and enriched beneficial bacterial community then synergistically promote the development of lamb small intestinal villi and the maturation of intestinal structure, manifested as higher villus height and villus-to-crypt ratio. At the host metabolic level, supplementary feeding not only drove more efficient protein synthesis (as evidenced by increased serum ALB and UREA) and lipid utilization (as indicated by reduced serum TC) but also effectively maintained redox homeostasis through an enhanced antioxidant defense system (manifested as increased activities of CAT, SOD, and GSH-PX, alongside slightly elevated levels of oxidative products MDA and H_2_O_2_). This provided a robust physiological internal environment for efficient growth, ultimately directing the ingested nutrients more effectively toward growth deposition (Fig. 6). Therefore, early supplemental feeding comprehensively enhances lamb growth performance, body size development, and alleviates post-weaning growth restriction through the cascade effect of “microbiota-fermentation-gut-metabolism.”

## MATERIALS AND METHODS

### Experimental design and treatments

The animal trial was conducted at the Tan sheep farm of Ningxia Hongsibao Tianyuan Fine-Breed Sheep Farming & Propagation Co., Ltd. Sixty healthy single-born lambs with similar birth dates (within 3 days) and initial body weight (4.00 ± 0.50 kg) were selected and randomly divided into a control group, Trial Group I, and Trial Group II, with 20 lambs per group. All experimental lambs were sourced from the same core breeding flock and were bred under a controlled program designed to avoid inbreeding, in order to minimize the potential impact of genetic variation on the experimental outcomes. Control group (C group) lambs suckled freely, with other rearing management conditions consistent with the trial groups. Trial Group I lambs suckled freely and had free access to supplemental mixed feed (80% pellet + 20% alfalfa meal) from 10 days of age, using lamb supplemental feeding pens where lambs could freely enter and exit, but ewes could not enter the supplemental feeding area. Trial Group II lambs suckled freely and had free access to supplemental mixed feed from 10 days of age, and from 15 days of age, they were forcibly isolated in the supplemental feeding pen from 9:00 a.m. to 5:00 p.m. daily to freely consume the supplemental mixed feed (Fig. 1A). The 585 lamb concentrate supplement used in the trial was purchased from Yinchuan Zhengda Co., Ltd. (feed production license number: Ning Feed License [2019] 01011). The nutritional composition of the supplemental mixed feed is shown in Table S1. Throughout the trial period, all lambs had free access to water. Body weight was measured at 0, 15, 30, 45, and 60 days of age each morning before feeding using a scale (XK3190-A12E, Shanghai Yaohua Weighing System Co., Ltd., China) to calculate phase average daily gain. Body measurements (height, body length, chest circumference, chest depth, and chest width) were taken at 0, 15, 30, 45, and 60 days using a tape measure (280213t, Deli, China) and a measuring stick (BMR-CZ, Beimuxin, China). After weaning at 60 days, lamb weights were continuously monitored at 90, 120, 150, 180, and 365 days.

### Sample collection

During the trial phase, blood samples were collected from the jugular vein using 5 mL blood collection tubes after morning fasting on days 9, 12, 15, 18, 21, 30, 45, and 60. Serum was separated after standing at room temperature for 2 h, followed by centrifugation at 3,000 r/min for 15 min, and then stored at −20°C. At 60 days of age, four lambs with body weights close to the group average were selected from each of the C, TI, and TII groups. After 2 h of water deprivation and 24 h of feed deprivation, the lambs were humanely euthanized for tissue collection. Rumen fluid samples were collected, filtered through four layers of gauze, immediately frozen in dry ice, and stored at −80°C. Tissue samples (approximately 4 cm in length) were rapidly collected from the duodenum (approximately 10 cm distal to the pylorus), jejunum (approximately one-third distal to the ligament of Treitz), and ileum (approximately 10 cm proximal to the ileocecal valve), flushed with ice-cold physiological saline to remove luminal contents, and then fixed in 4% paraformaldehyde.

### Rumen fermentation parameters

Filtered rumen fluid pH was immediately measured using a handheld pH meter (PHB-3, Sanxin, China). Rumen fluid NH_3_-N concentration was determined using the alkaline sodium hypochlorite-phenol method. TVFA concentration in rumen fluid was analyzed by gas chromatography, and the proportion of each VFA to TVFA and the acetate-to-propionate ratio were calculated. Rumen fluid, thawed at 4°C for 2 h, was centrifuged at 4°C at 12,000 rpm for 10 min. Then, 1.5 mL of supernatant was transferred to a 2 mL centrifuge tube, 200 µL of 25% metaphosphoric acid (25 g metaphosphoric acid dissolved and made up to 100 mL with double-distilled water) was added, and incubated overnight at 4°C. The incubated samples were centrifuged at 4°C at 13,000 rpm for 15 min, and 1 mL of supernatant was transferred to 1.5 mL of 0.2458% crotonic acid (0.2458 g crotonic acid dissolved and made up to 100 mL with double-distilled water) and incubated at 4°C for 4 h. Then, 800 µL of the above mixture was transferred to a gas chromatography vial for analysis. Short-chain fatty acids were determined using an Agilent gas chromatograph (7890A GC system, Agilent, USA) with the program set as: injector and FID detector temperature set at 200°C, column temperature held at 100°C for 1 min, then raised to 200°C and held for 6 min, finally running for 1 min.

### Hematoxylin and eosin staining

After fixation, duodenal, jejunal, and ileal tissue samples were routinely embedded in paraffin and sectioned at 4 μm thickness. Sections were deparaffinized in xylene, hydrated through a graded ethanol series, and stained with hematoxylin and eosin: nuclei were stained with hematoxylin for 5 min, differentiated in hydrochloric acid ethanol, and blued in running water; cytoplasm was stained with eosin for 1 min, followed by dehydration in ethanol, clearing in xylene, and mounting with neutral balsam. The morphological structure of each intestinal segment was observed under a light microscope. Villus length and crypt depth of the duodenum, jejunum, and ileum were measured. The ratio of villus length to crypt depth was calculated based on the measured data. The longest villus length and deepest crypt depth in each field of view were measured using an ocular micrometer, with five measurements per indicator.

### Biochemical indicators

Serum growth hormone and insulin-like growth factor 1 concentrations were measured using commercial enzyme-linked immunosorbent assay kits (GH: Shanghai Bangyi Biotechnology, BYE93037; IGF-1: Shanghai Bangyi Biotechnology, BYE80149), strictly following the manufacturer’s instructions. The procedure included incubation, addition of biotinylated detection antibody, horseradish peroxidase-labeled streptavidin, and TMB substrate development. Absorbance was measured at 450 nm wavelength using a fully automated microplate reader (BioTek Synergy H1, Agilent, USA), and concentrations were calculated based on the standard curve. Other biochemical indicators, including total protein, triglycerides, urea (UREA), total cholesterol, glucose, and albumin (ALB), were determined using an automatic biochemical analyzer (BS-420, Shenzhen Mindray Bio-Medical Electronics Co., Ltd., China) and corresponding reagent kits.

### Oxidative stress and antioxidant indicators

Serum superoxide dismutase (SOD), catalase (CAT), glutathione peroxidase (GSH-PX) activities, and malondialdehyde and hydrogen peroxide contents were measured using colorimetric kits (all purchased from Shanghai Bangyi Biotechnology Co., Ltd.). The kit codes for SOD, CAT, GSH-PX, MDA, and H_₂_O_₂_ detection were BYE93043, BYE80434, BYE81518, BYE80435, and BYE93024, respectively. All operations were performed according to the kit instructions, and results were read using a microplate reader (ELx800, BioTek, USA). All measured values were corrected using serum total protein concentration determined by the BCA method (51), with final results expressed as U/mL.

### DNA extraction and PCR amplification

DNA was extracted from centrifuged rumen fluid samples according to the manufacturer’s instructions. DNA concentration and purity were determined using a NanoDrop2000 UV-Vis spectrophotometer. DNA quality was assessed using 1% agarose gel electrophoresis. The V3-V4 region of the microbial 16S rRNA gene was amplified using universal bacterial primers 338F (5′-ACTCCTACGGGAGGCAGCAG-3′) and 806R (5′-GGACTACHVGGGTWTCTAAT-3′), with a target product of 468 bp, using a PCR instrument (ABI GeneAmp 9700). The PCR reaction system was as follows: a total of 20 µL reaction system, including 4 μL 5× Taq buffer, 2 μL dNTP mixture (2.5 mmol/L), 0.4 µL Taq DNA polymerase (5 U/µL, Mg²^+^ plus), 0.8 μL each of forward and reverse primers (5 μmol/L), 1 µL genomic DNA (10 ng/µL), and the remainder made up with ultrapure water. The PCR program was as follows: 95°C pre-denaturation for 30 s; 35 cycles of 95°C denaturation for 30 s, 50°C annealing for 30 s, and 72°C extension for 45 s; and final extension at 72°C for 10 min. PCR products from the same sample were mixed and detected by 2% agarose gel electrophoresis. PCR products were recovered using the AxyPrep DNA Gel Extraction Kit (AXYGEN Company), eluted with Tris-HCl, and detected by 2% agarose electrophoresis. Based on preliminary electrophoresis quantification results, PCR products were quantified using the QuantiFluor-ST Blue Fluorescence Quantification System (Promega Company), then mixed in corresponding proportions according to the sequencing requirements of each sample.

#### Library construction

Illumina official adapter sequences were added to the outer ends of the target regions via PCR. PCR products were recovered by gel extraction using a gel recovery kit, eluted with Tris-HCl buffer, detected by 2% agarose electrophoresis, and denatured with sodium hydroxide to produce single-stranded DNA fragments.

### 16S rRNA sequencing analysis

Adapter sequences of DNA fragments were complementary to the embedded base sequences on an Illumina flow cell (Illumina, San Diego, CA, USA; HiSeq 2500 flow cell), which was used for cluster generation. Sequencing was performed by Shanghai Majorbio Bio-Pharm Technology Co., Ltd. (Shanghai, China), using their full-service 16S rRNA sequencing workflow. Using DNA fragments as templates and the fixed base sequences on the chip as primers for PCR synthesis, target DNA fragments to be tested were synthesized on the chip; after denaturation and annealing, the other end of the DNA fragment on the chip randomly complemented another nearby primer and was also fixed, forming a “bridge”; PCR amplification produced DNA clusters. DNA amplicons were linearized into single strands. Modified DNA polymerase and dNTPs with four fluorescent labels were added, synthesizing only one base per cycle. The reaction plate surface was scanned with a laser to read the type of nucleotide polymerized in each round of reaction for each template sequence. The “fluorescent group” and “termination group” were chemically cleaved, restoring the 3′ end stickiness, and nucleotide polymerization continued. The fluorescence signals collected in each round were statistically analyzed to obtain the sequence of the template DNA fragments. After sample splitting of the PE reads obtained by Illumina sequencing, paired-end reads were first quality-controlled and filtered based on sequencing quality and simultaneously spliced based on the overlap relationship between paired-end reads to obtain optimized data after quality control and splicing. Then, sequence denoising methods, including DADA2 (52) and Deblur (53), were used to process the optimized data to obtain amplicon sequence variant representative sequences and abundance information. Subsequent data analysis and processing were performed using the Majorbio Cloud Platform (https://v.majorbio.com/project-center/task).

### Statistical analysis

Average daily feed intake, average daily gain, and economic benefits per pen during the full feeding period were calculated based on body weight and feed consumption data. Economic benefits were calculated as lamb weight gain income minus feed costs in the trial year, expressed in RMB. Differences in growth performance traits (body weight, daily gain, body height, body length, chest width, chest depth, and chest circumference), rumen fermentation parameters (pH, NH_3_-N, TVFA, etc.), intestinal development (villus length, crypt depth, etc.), serum biochemical indicators (GH, IGF-1, TP, TG, UN, etc.), and serum oxidative stress indicators (SOD, CAT, MDA, H_₂_O_₂_, etc.) among the three groups were tested using one-way ANOVA, with Student’s *t*-test for comparisons between two groups. Differentially abundant taxa between groups were identified using the Kruskal-Wallis test, with the Wilcoxon rank-sum test for comparisons between two groups, and *P*-values were corrected using the false discovery rate value < 0.05. Correlations between bacterial abundance, rumen fermentation parameters, intestinal development, serum metabolites, and growth performance were tested using Spearman’s rank correlation coefficient. *P* < 0.05 was represented by * and different letters, *P* < 0.01 by **, *P* < 0.001 by ***, and *P* < 0.0001 by ****.

## Data Availability

Sequencing data have been deposited in the NCBI Sequence Read Archive (SRA) under accession number PRJNA1357076. All data generated or analyzed during this study are available from the corresponding author upon reasonable request.

## SUPPLEMENTAL MATERIAL

Supplemental material aem.02421-25-s0001.docx Table S1; Fig. S1 and S2.

ASM does not own the copyrights to Supplemental Material that may be linked to, or accessed through, an article. The authors have granted ASM a non-exclusive, world-wide license to publish the Supplemental Material files. Please contact the corresponding author directly for reuse.
