# Supplementary material for: Early supplemental feeding improves post-weaning growth restriction in lambs via the gastrointestinal-metabolic axis
Source: Appl Environ Microbiol. 2026 May 15;92(6):e02421-25. doi: 10.1128/aem.02421-25 (PMC13274353; doi:10.1128/aem.02421-25)
Supplement: Supplemental material — Table S1; Fig. S1 and S2. [file aem.02421-25-s0001.docx]

**Supplementary Information**
**Supplementary Table 1.** Chemical composition of supplemental mixed feed (air-dry basis)

| Tems | Nutrient levels |
| --- | --- |
| Dry Matter | 90.40 |
| Metabolizable Energy（MJ/kg） | 11.90 |
| Crude Protein | 16.39 |
| Ether Extract | 3.30 |
| Ash | 8.43 |
| Neutral Detergent Fiber | 22.40 |
| Acid Detergent Fiber | 14.00 |
| Ca | 0.94 |
| P | 0.55 |

All components except metabolizable energy are expressed as percentage of air-dry feed mass. Metabolizable energy is expressed as MJ/kg of air-dry feed. All nutrient levels were determined by laboratory analysis.


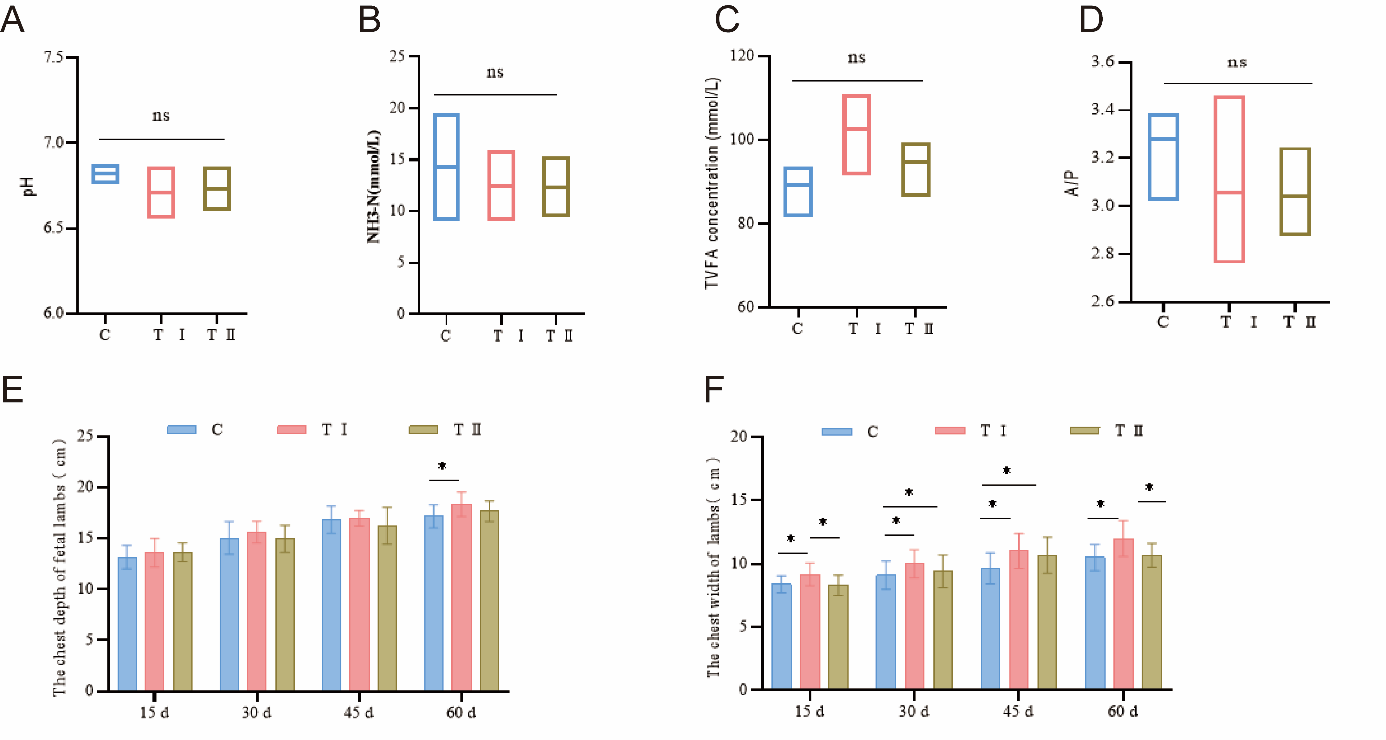


**Supplementary Figure 1. Effects of different early supplemental feeding regimens on lamb growth performance and rumen fermentation parameters at 60 days of age.
(A)** Rumen fluid pH in lambs. **(B)** Rumen fluid ammonia nitrogen (NH₃-N) concentration in lambs. **(C)** Rumen fluid total volatile fatty acid (TVFA) concentration in lambs. **(D)** Acetate to propionate ratio (A/P) in rumen fluid of lambs. **(E)** Chest depth of lambs. **(F)** Chest width of lambs. Statistical comparisons were conducted using Student's t-test. Data are presented as mean ± SEM. Significance levels: n.s. P > 0.05, *P < 0.05.


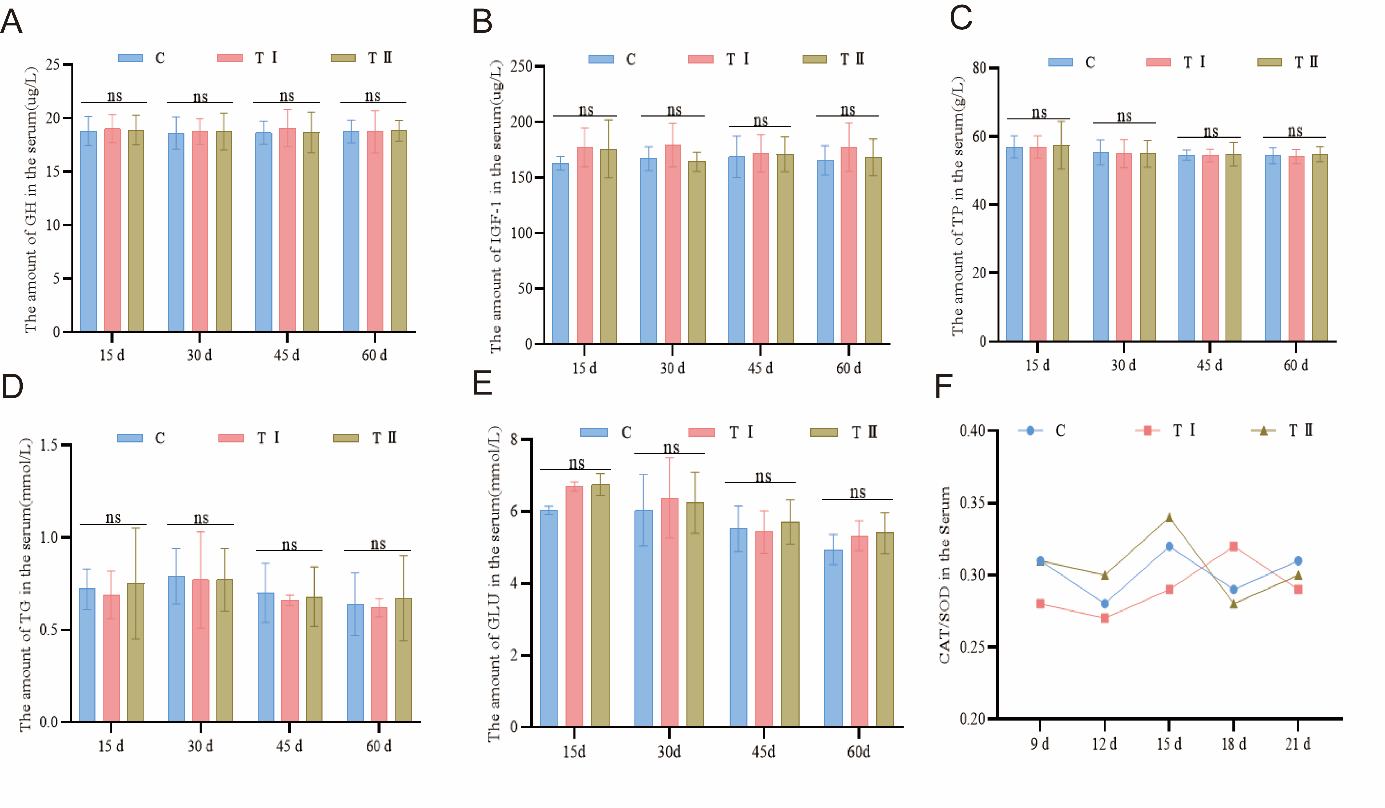


**Supplementary Figure 2. Effects of different early feeding regimens on serum biochemistry, oxidative stress and antioxidant capacity in lambs**
**(A)** Serum growth hormone (GH) concentration. **(B)** Serum insulin-like growth factor-1 (IGF-1) concentration. **(C)** Serum total protein (TP) concentration. **(D)** Serum triglyceride (TG) concentration. **(E)** Serum glucose (GLU) concentration. **(F)** Trend in the serum CAT/SOD ratio. Statistical comparisons were conducted using Student's t-test. Data are presented as mean ± SEM. Significance levels: n.s. P > 0.05, *P < 0.05.
